# Identification of an oncological clinical pathway through questionnaires to health professionals

**DOI:** 10.1186/s12913-023-09964-w

**Published:** 2023-09-20

**Authors:** Mario Forrester, Luiza Breitenfeld, Miguel Castelo-Branco, Jorge Aperta

**Affiliations:** 1 Faculty of Health Sciences Universidade Da Beira Interior, Av. Infante D. Henrique, Covilhã, 6200-506 Portugal; 2 Sousa Martins Hospital, Avenida Rainha Dona Amélia, Guarda, 6300-858 Portugal

**Keywords:** Clinical pathways, Value-based healthcare, Oncology, Patient management

## Abstract

**Background:**

Clinical Pathways in Oncology can benefit patients using organized interventions to standardize and increase care efficiency. Healthcare systems should have tools to identify their oncological clinical pathways for a better institutional organization to reduce mortality rates and contain costs without compromising quality. Our objective is to determine the regional Oncology Clinical Pathway from a first basic hypothesis using questionnaires directed to healthcare professionals considered key deciders within the Pathway.

**Methods:**

Study design consisted of data analysis of two structured region-wide questionnaires; built using available literature on Oncology Clinical Pathways, in a Portuguese Healthcare context and pre-tested in a focus group of key deciders (Physicians and nurses with management functions) from which a design was created. Queries analyzed the patients: tumor staging at service arrival; time intervals on tumor suspicion/diagnosis confirmation and diagnosis/first treatment; referral pathway; diagnostic networks and patient Follow-up. One questionnaire was sent to key deciders directly involved with Oncology patients at a Regional Hospital. 15 physicians and 18 nurses of this sample answered the questionnaire (approx. response rate = 67%). Another questionnaire sent to healthcare professionals in Primary Healthcare Centers yielded response rate 19.2%, *N* = 29 physicians and 46 nurses. Finally, we performed a descriptive analysis and a Cronbach Alpha reliability analysis.

**Results:**

Our findings reveal: different appreciations of tumor staging at arrival in Primary Healthcare Centers and Regional Hospitals (the latter receiving more metastatic cases); approximately 4 weeks between tumor suspicion-diagnostic and divided opinions regarding diagnostic-treatment time intervals. Primary Healthcare Centers depend on private laboratories for diagnostics confirmation, while the Hospitals resolve this locally. Referral pathways indicate almost half of the patients being sent from primary healthcare centers to National Reference Hospitals instead of a Regional Hospital. Patient follow-up is developed throughout the institutions, however, is more established at Regional Hospitals. As patients advance through the Oncology Clinical Pathway and toward treatment stages the number of healthcare professionals involved reduce.

**Conclusion:**

Our questionnaires enable us to understand the real pathway between the different institutions involved and the main entry points of the patients into the Oncology Clinical Pathway.

**Supplementary Information:**

The online version contains supplementary material available at 10.1186/s12913-023-09964-w.

## Background

Oncological care is an extremely complex matter that requires the involvement of various healthcare professionals to provide the attention needed to improve the patient’s quality of life. Diagnostic and therapeutic measures must be effective, safe, timely, patient-centered, and of high quality. However, without good coordination of care, patients, caregivers, and families may receive fragmented health services from multiple providers, leading to suboptimal outcomes, poor medication reconciliation, inadequate sharing of clinical information, duplicated processes, and avoidable hospital admissions [1–3].

Clinical Pathways (CPs) are developed through evidence-based recommendations and supporting evidence, creating a standardized, multidimensional roadmap for longitudinal care with key milestones and decision points for multiple episodes of care. This roadmap, which we named as the Oncological Care Pathway (OCP), is essential for ensuring organized interventions for managing care processes, as defined by The American Society of Clinical Oncology (ASCO). The identification of an oncological pathway aims to enhance care coordination and continuity across various healthcare sectors and disciplines [4–10].

According to two systematic reviews, the relevance of clinical pathways has been increasing since the early 2000s, with most of the work being published since 2010. There has been a steady growth and a significant increase in publications after 2012 [11]. Among medical specialties, oncology and cardiology are the two leading specialties with the highest number of publications. Also, the United States and China are the countries with the highest number of publications on clinical pathways. In the context of our study, Portugal only has one study included in this systematic review, which highlights the need to increase the number of studies on clinical pathways in Portugal [12].

Moreover, the developing and analysis of a CP within a specific healthcare context requires empirical analysis, owing to the diversity of healthcare systems and institutions, each with their distinctive overall and internal logistics. Both reviews acknowledge a limitation in the analyzed studies due to their broad scope, which in turn restricts thorough discussion and raises methodological concerns. Nonetheless, these reviews offer valuable insights into the multiple ways CPs can deliver pertinent empirical information to healthcare planners, managers, and clinical practitioners. Their outcomes could have important implications for policymakers, decision makers, managers, and researchers, as they hold the potential to establish an international consensus, finally facilitating comparisons of care pathway improvement programs [11, 12].

To contextualize the importance of our work, several studies of clinical pathways for Oncology and other diseases have been developed in countries like USA, China, France, Norway, Scotland, England, New Zealand, The Netherlands, Hungary, Italy, South Korea, Canada, and Denmark, as shown in Supplementary table 1 (Additional file 1: Annex 1) [9, 13–26].

In the Portuguese healthcare context, patients’ first point of contact is usually with a general practitioner at a nearby Primary Healthcare Center (PHC) based on their residential or work location. However, in urgent situations, patients may bypass this step and go directly to emergency departments seeking specialized care. Patients who are covered by health subsystems can also go directly to private hospitals and specialists approved by their schemes, with an option to further refer back to the National Health System [27]. Furthermore, the Portuguese healthcare system is universal and tends to be free of charge. It offers several levels of access, including primary care, mainly provided at local healthcare centers, and advanced care for acute situations at regional hospitals (with fewer specialties), General Central hospitals, and specialized Central Hospitals. In addition, citizens have the option to choose complementary private care.

Regarding Oncology in Portugal, the healthcare system consists of three care platforms (A, B, and C). C Platforms integrate Regional Hospitals (RHs), B Platforms integrate Central Hospitals and Regional Centers of the Portuguese Institute of Oncology, and A platforms integrate the Portuguese Institute of Oncology and are responsible for cancer care politics in Portugal, by promoting investigation and treating the most complex cases demanding advanced techniques and treatments [28].

According to the OECD/European Observatory on Health Systems and Policies, Portugal has approximately 58,000 new cases of cancer each year. The most common cancer sites among men are prostate, colorectal, and lung, while breast cancer is the leading tumor among women, followed by colorectal and lung cancer. In 2016, Portugal launched a National Cancer Plan that focuses on promoting prevention, early diagnosis, and treatment, while ensuring that all citizens have equitable access to cancer care. The plan aims to address the expected 30,000 cancer deaths per year since 2020 [29].

**Fig. 1:**
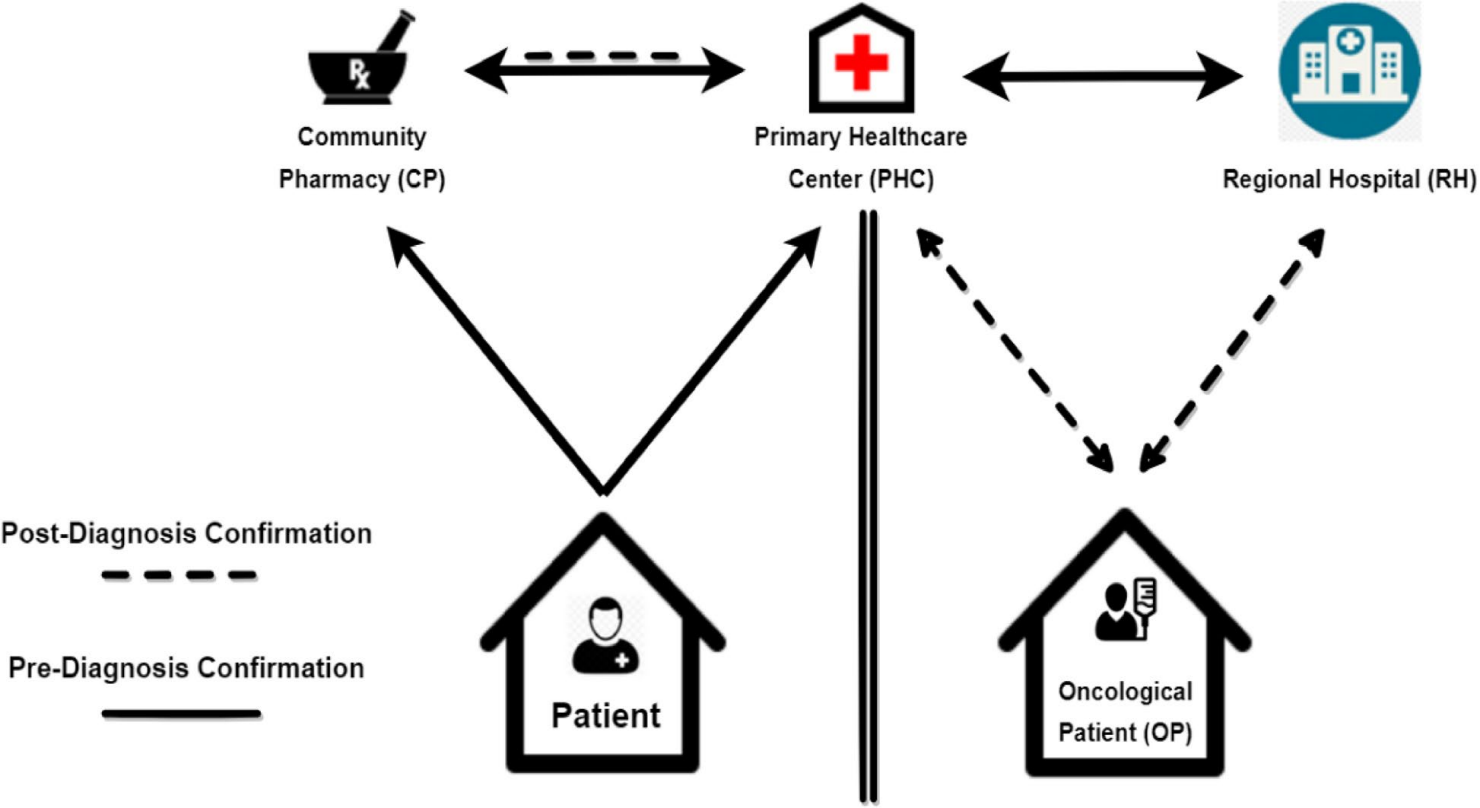
First hypothesis of a Regional Oncological Clinical Pathway. Patient Pre-Diagnostic Confirmation Pathway order: 1) CP and/or PHC, 2) RH. OP Post-Diagnostic Confirmation Pathway order: 1) RH, 2) PHC, 3) CP

Since the main participants in this study are professionals from hospitals, primary healthcare centers, and possibly community pharmacies, we have developed a hypothesis for designing an OCP for a region distant from Portugal’s major urban centers, as shown in Fig. 1. Our approach considers the region’s health partners, as recommended by Portuguese healthcare experts, and aims to create an OCP that is both geographically close and economically feasible, while also enhancing the quality of life for patients. Given that nurses and physicians are the primary healthcare professionals responsible for guiding patients throughout their pathological process, we have created a set of questionnaires to characterize the actual OCPs as perceived by healthcare professionals who are likely to be involved with cancer patients, such as physicians and nurses from hospitals and primary healthcare centers [30, 31].

Analyzing the differences and similarities in healthcare organization can show a starting plan for in-depth research possibilities to improve efficiency and patient related outcomes [32]. Therefore, our work meant to determine the tumor staging at PHCs and RH arrival, the time intervals within the OCP stages, where diagnostics analysis are done, how the referral network is organized, and if patient follow-up is a common practice.

## Methods

Our study design consists of data analysis of two structured region-wide questionnaires sent by email and/or physically delivered. The questionnaires for this descriptive study were built using available literature on OCPs, adapted to a Portuguese Healthcare context using experts’ insight, and pre-tested in a focus group that integrates key deciders (physicians and nurses with management functions). Queries analyzed the patients: tumor staging at service arrival; time intervals on tumor suspicion/diagnosis confirmation and diagnosis/first treatment; referral pathway; diagnostic networks; and patient follow-up. Tumor types included in this analysis were selected based on the RH treatment capacity.

We started our study by validating the questionnaire through a pre-test. The pre-test consisted of three physicians and three nurses working at a RH, additionally, four physicians and two nurses from PHCs. Based on the focus group answers we concluded that our questionnaires would need an approximate time of 30 min to be answered, more categories for the probable and confirmed diagnosis questions in the hospital questionnaire, and a “*not applicable*” category in both questionnaires to exclude responders not involved with certain types of patients and/or tumors, moreover, language corrections were also addressed by Portuguese experts.

**Fig. 2:**
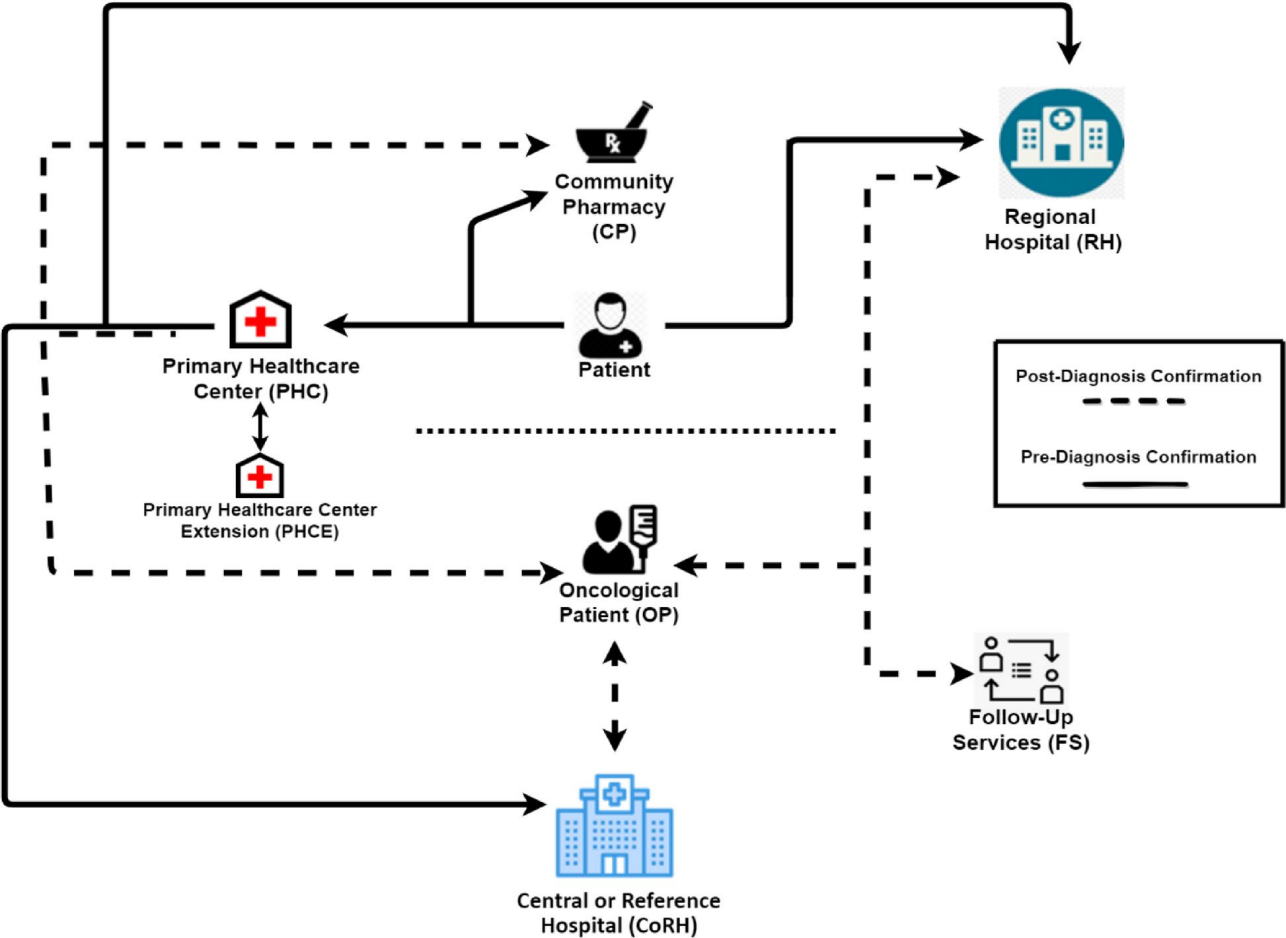
Oncological Clinical Pathway identified after first test. Patient Pre-Diagnostic Confirmation Pathway order: 1) PHC, 2) RH, 3) CoRH, 4) CP. OP Post-Diagnostic Confirmation Pathway order: 1) FS, 2) PHC, 3) RH, 4) CoRH, 5) CP

The pre-test enables us to generate a second design for an OCP, as per Fig. 2. This new design differs from the one in Fig. 1, as the experts’ insight and the focus group revealed more involved institutions and services in the regional OCPs in Portugal.

This new design has similar organization, as the one proposed by Brenne et al. for palliative care in Norway, and could help picture the different pathways available to cancer patients as done in other studies for other health conditions like acute stroke reperfusion delivery [14, 33].

Afterwards, the questionnaire in Additional file 1: Annex 2 was sent to physicians and nurses directly involved with oncology patients at the Regional Hospital (approximately 49 health professionals). Fifteen physicians and eighteen nurses of this sample answered the questionnaire (Approx. response rate = 67%). The questionnaire in Additional file 1: Annex 3 was sent as well to healthcare professionals of the Primary Healthcare Centers (approximately 389 health professionals). Twenty-nine physicians and forty-six nurses returned the questionnaire (Approx. response rate = 19,2%). The sample size in our study was not calculated as it was all the available population that fitted the inclusion criteria; however, this could show how knowledgeable our participants are regarding the regional OCPs.

Moreover, the study inclusion criteria were being a nurse or physician with management functions of oncological patients at hospitals and all the nurses and physicians at healthcare centers; working within the Portuguese public healthcare system; and being employed by the local healthcare unit where the study was performed. 

The final versions of the questionnaires were applied confidentially (via online through Google Forms and physically delivered to nurses and physicians) in a regional health unit that integrates two regional hospitals and 14 Healthcare Centers in a Portuguese interior region (supporting a population of 138 211 habitants). This collection method proved to be more effective and adaptable to our participants’ schedules, enabling us to conduct the study for a 4-month period from January to April 2021, with regular reminders sent via email or telephone every 2 weeks. The digital format of the questionnaire facilitated faster data collection and development of datasets, which was a more efficient process than the traditional method that required traveling throughout the region and manual data entry.

Before collecting data, we obtained the necessary permissions from the local health unit’s Ethics Committee. The questionnaires were adapted to the region and context in which they were applied, therefore, we suggest that any further use of these questionnaires for analyzing healthcare systems or institutions be tailored accordingly. Results were analyzed through a descriptive analysis and a Cronbach Alpha reliability analysis using IBM SPSS Statistics 25 statistical software.

The first questionnaire (Additional file 1: Annex 2) was focused on identifying patient OCP characteristics at a RH level and the second one (Additional file 1: Annex 3) intended the same objective in a PHC context. Both questionnaires shared questions for a horizontal analysis and included questions for a vertical analysis.

To determine the initial steps of the patients in the OCP, we analyzed questions 2, 3, 4, and 5 of the RH questionnaire (shown in Additional file 1: Annex 2) and questions 3, 4, 5, and 7 of the PHCs questionnaire (Additional file 1: Annex 3) in a horizontal manner. These questions are crossed information checklists that allow responders to select the options that best that best apply to the type of tumor being analyzed. This information could help determine how much time is needed for patients to start their treatment once properly diagnosed.

To determine the follow-up steps of the OCP, we conducted an analysis of question 9 from RH questionnaire and question 11 from PHC questionnaire, both of which consisted of closed-ended questions. The remaining questions analyzed were specific from each questionnaire (vertical analysis) and were of multiple choice. Their objective is to determine how the OCP network is organized.

The study intended to collect quantitative data that could help identify and analyze the characteristics of the OCP of this region. Quantitative data was treated descriptively and analyzed using simple and easy to understand Figures. We performed a Cronbach Alpha reliability analysis in different sets of questions on our horizontal analysis (questions 2, 3, 4, and 5 of the RH questionnaire and questions 3, 4, 5, and 7 of the PHCs questionnaire).

The descriptive analysis will then be used to further analyze the proposed OCP design (Fig. 2) and suggest modifications. To best reflect the insight and the experience of our participants, we excluded all the “not applicable” answers from our study thus analyzing the answers of those healthcare professional that overview certain types of patients and tumors.

## Results

### Tumor staging

**Fig. 3:**
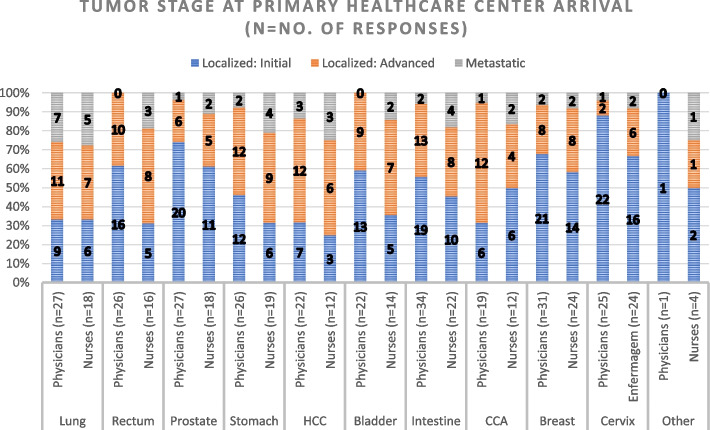
Tumor stage at Primary Healthcare Center Arrival. HCC: Hepatocellular Carcinoma; CCA: Cholangiocarcinoma

**Fig. 4:**
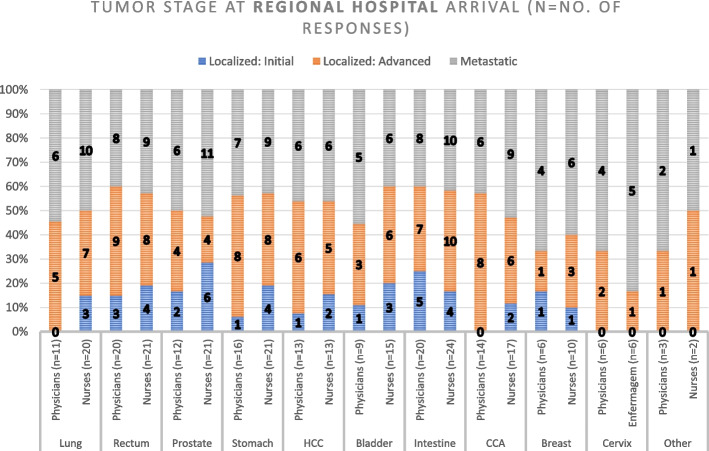
Tumor stage at Regional Hospital Arrival. HCC: Hepatocellular Carcinoma; CCA: Cholangiocarcinoma

Regarding the first question in our analysis, PHC staff (physicians and nurses) considered that most of the oncology patients they receive, arrive at Initial and Advanced stages, as shown in Fig. 3. When asked the same question, RH staff (physicians and nurses) stated that the majority of their patients arrive at their services in Advanced and Metastatic cases with fewer Initial cases, as shown in Fig. 4. The questions used to measure Tumor Staging at arrival in PHCs and at a RH consisted of 11 items both. The scale had a high level of internal consistency as determined by their Cronbach alpha values of 0,955 and 0,885 for Figs. 3 and 4, respectively.

### Tumor suspicion and diagnostic confirmation time interval

**Fig. 5:**
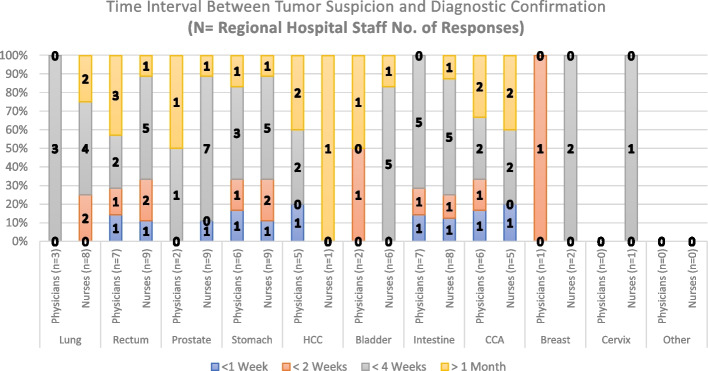
Time interval between tumor suspicion and Diagnosis Confirmation (Regional Hospital Staff Responses). HCC: Hepatocellular Carcinoma; CCA: Cholangiocarcinoma

**Fig. 6:**
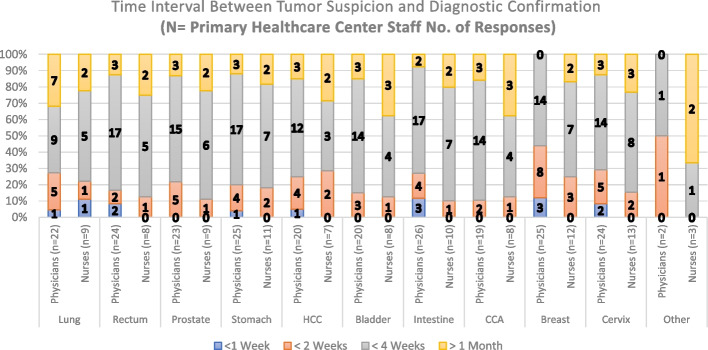
Time interval between tumor suspicion and Diagnosis Confirmation (Primary Healthcare Center Staff Responses). HCC: Hepatocellular Carcinoma; CCA: Cholangiocarcinoma

The second question from our questionnaire intends to determine an approximate time between tumor suspicion and diagnosis based on the daily appreciation of the responders. Thus, Fig. 5 shows a majority of answers from the RH staff, stating an approximate time interval for most treated tumors, of less than 4 weeks with several cases taking more than 1 month on diagnostic confirmation. Concerning the PHC staff, Fig. 6 shows higher percentages of answers in which the approximate time interval of diagnosis is less than 4 weeks. Questions employed to measure Time interval appreciation between Tumor suspicion and Diagnosis in PHCs and the RH consisted of also 11 items both. The scale had a high level of internal consistency as determined by their Cronbach alpha values of 0,843 and 0,969 for Figs. 5 and 6, respectively.

### Diagnosis and first treatment time interval

**Fig. 7:**
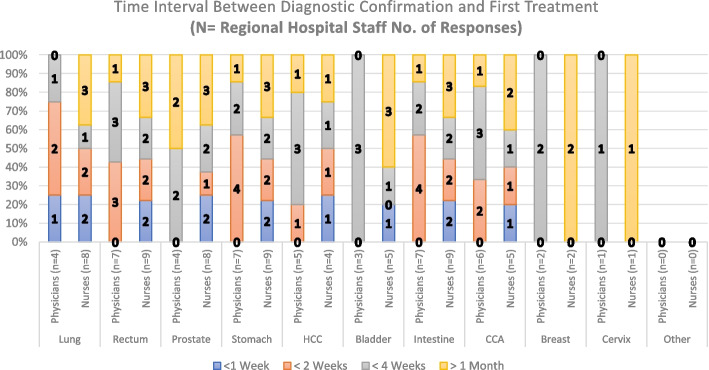
Time interval between Diagnosis Confirmation and First Treatment (Regional Hospital Staff Responses). HCC: Hepatocellular Carcinoma; CCA: Cholangiocarcinoma

**Fig. 8:**
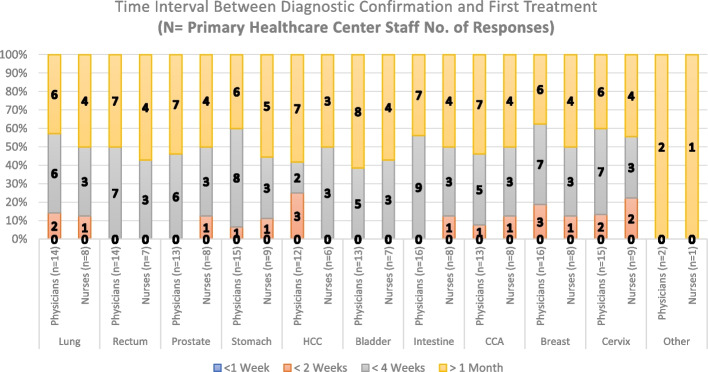
Time interval between Diagnosis Confirmation and First Treatment (Primary Healthcare Center Staff Responses). HCC: Hepatocellular Carcinoma; CCA: Cholangiocarcinoma

Regarding the approximate time patients wait to get their first treatment (surgery and/or medication), Fig. 7 shows responses from the RH staff in all the time interval options. However, most of the answers for Diagnosis/Treatment intervals are distributed in the < 2 weeks, < 4 weeks, and > 1-month categories. As per the PHC staff, Fig. 8 shows the approximate Diagnosis/Treatment time interval is mostly more than 1 month and/or less than 4 weeks. Questions employed to measure Time interval appreciation between Diagnosis and First Treatment in PHCs and the RH consisted of 11 questions both. The scale had a high level of internal consistency as determined by their Cronbach alpha values of 0,907 and 0,978 for Figs. 7 and 8, respectively.

### Primary healthcare centers referral pathway

**Fig. 9:**
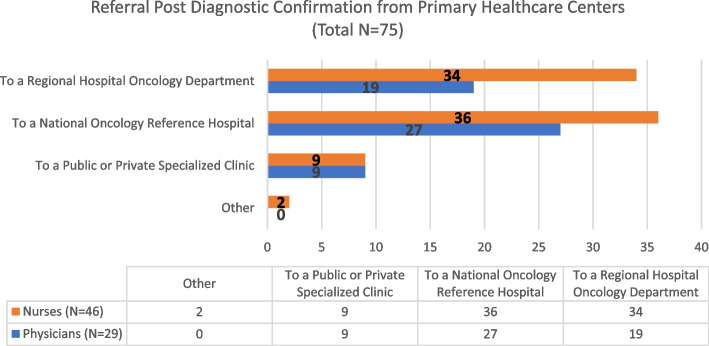
Local of Post Diagnostic Confirmation Referral at Healthcare Centers

To describe the OCP available to PHCs within the local health unit, responders selected from the questionnaire which institutions (Hospital, Clinics, others) are part of their referral network. Thus, Fig. 9 intends to determine which are the following steps once patients with tumor suspicion attend their local Healthcare Center. Most of the oncology patients are referred to a Central or Reference Oncology Hospital (approximately 200kms away from the RH) or the Regional Hospital Oncology Department. Furthermore, from the 75 PHC responders, 49 (65,3%) indicated coordination between the institutions from Fig. 9, with only 2 (2,7%) stating the level of coordination was excellent, 33 (44,0%) good, 21 (28%) reasonable, 12 (16%) insufficient, and 7 (9,3%) did not answer (as per questions 9 and 10 in Additional file 1: Annex 3).

**Fig. 10:**
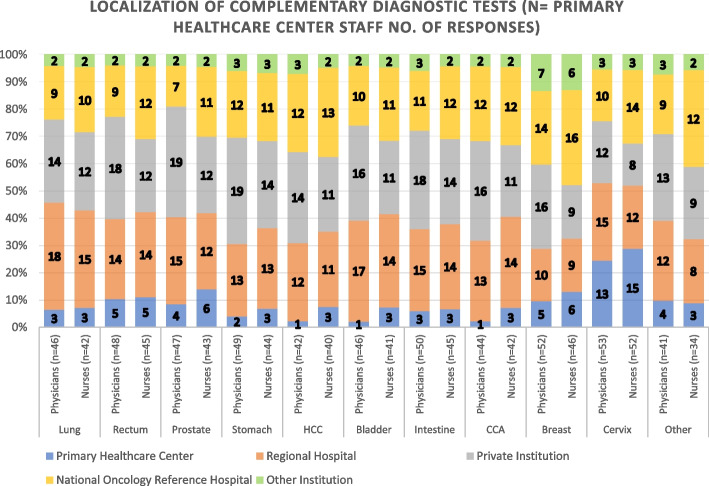
Local of Complementary Diagnostic Tests used by Primary Healthcare Centers. HCC: Hepatocellular Carcinoma; CCA: Cholangiocarcinoma

Complementary diagnostics are also part of the OCP available to PHCs. Our questionnaire intended to know which institutions conform the diagnostic network in our studied group. Most responders agree in the referral of their patients (for diagnostic purposes) to the Regional Hospital, Private laboratories, and/or Oncology Reference Institution, as shown in Fig. 10. Additionally, a large percentage of cervix tumors are diagnosed locally at the PHCs. Questions used to measure where complementary diagnostic tests are carried out according to PHCs responders consisted of 11 items. The scale had a high level of internal consistency as determined by their Cronbach alpha value of 0,972 for Fig. 10.

### Hospital diagnostic network

**Fig. 11:**
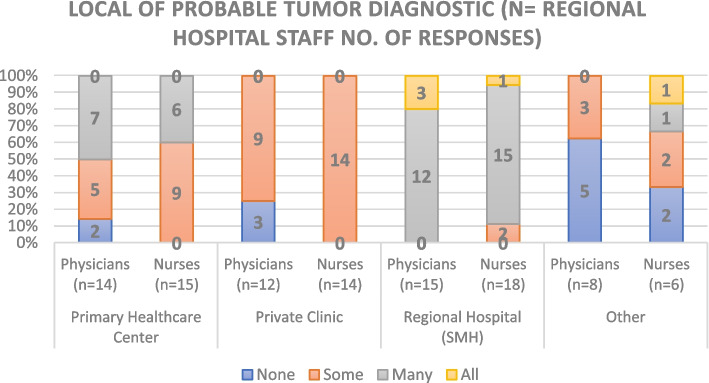
Local of Probable Tumor Diagnostic according to Regional Hospital Staff

**Fig. 12:**
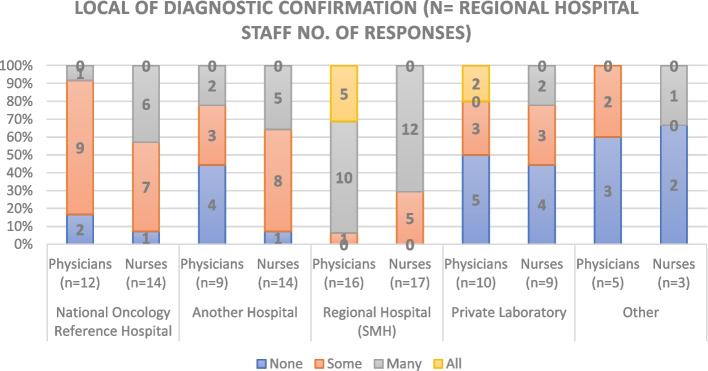
Local of Diagnostic confirmation according to Regional Hospital Staff

Hospital responders agreed that the probable tumor diagnosis of most of their patients (Fig. 11) is done at their institution, followed by PHCs, private centers, and other institutions. Diagnosis confirmation (Fig. 12), as stated by this group, occurs mainly at a RH level, followed by diagnostics done at reference hospitals, other regional hospitals, and private laboratories.

**Fig. 13:**
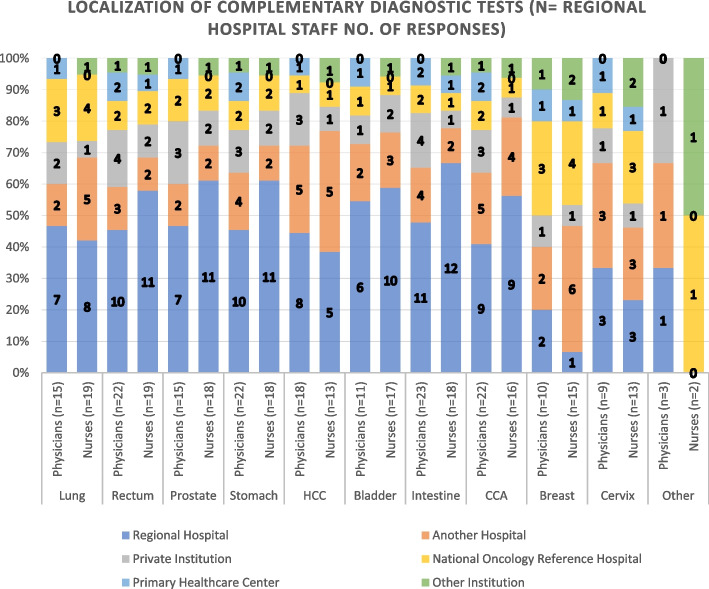
Local of Complementary Diagnostic Tests used by Regional Hospital Staff. HCC: Hepatocellular Carcinoma; CCA: Cholangiocarcinoma

Consequently, Fig. 13 also shows higher percentages of RH staff members responses agreeing that most of the complementary diagnostic tests are done at the RH. Questions used to measure where complementary diagnostic tests are carried out according to hospital responders consisted of 11 items. The scale had a high level of internal consistency as determined by their Cronbach alpha value of 0,944 for Fig. 13.

### Patient follow-up and referral to other hospitals

**Fig. 14:**
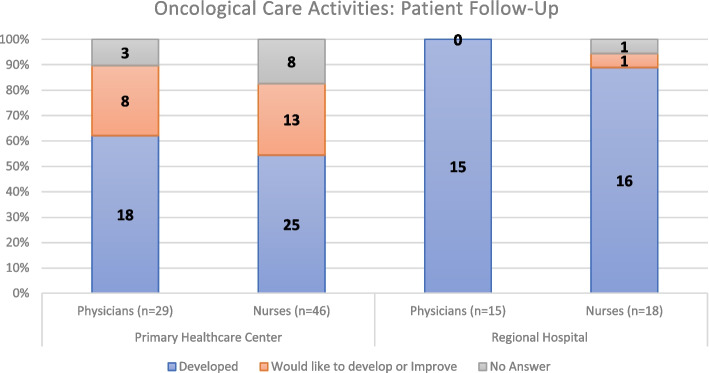
Cancer Patient Follow-up at the local health unit

Regarding Patient Follow-up (as regular appointments for cancer care and other comorbidities), Fig. 14 indicates most responders (physicians and nurses) at PHCs in the region carry out periodical follow-ups with their oncology patients, likewise, all physician responders at the RH as well as most nurse responders develop this activity. However, the proportion of nurses and physicians implementing this activity is higher at Hospital levels.

Patient referrals to other hospitals showed: from 33 hospital responders, 3 (9,1%) stated not referring any patients at all, 13 a few (39,4%), 15 (45,5%) some, and 0 referring all their patients. One physician (3%) stated he refers both a few and some of his patients and there was a nurse (3%) that did not answer (as per question 8 in Additional file 1: Annex 2). Hospital responders did not indicate referring large quantities of their cancer patients to other hospitals.

## Discussion

The results of this study reveal that within this interior region integrated by 2 joined Regional Hospitals and 14 Primary Healthcare Centers there are different approaches between Hospitals and Healthcare Centers regarding several steps of the OCP. Therefore, CPs methods should be used to standardize health care processes [34].

Our analysis of tumor staging at service arrival revealed discrepancies between PHCs and RHs in the region of study. While most PHC responders reported receiving patients with Localized Initial and Advanced Localized Tumors, RH responders also reported a high number of patients with Advanced Localized and Metastatic Tumors. These findings raise questions about the factors underlying such differences and suggest the need for further investigation to identify potential areas for standardization of cancer care processes in the region.

Published studies concerning barriers for tumor staging indicate several possibilities. First, patient mediated factors such as lack of knowledge, fear of the disease and time constraints can be associated with late stage presentations [35]. Second, patient navigation is often complex resulting in unnecessary delays, consequently, the type of physician a patient contacts initially will play an important role on timely staging [36, 37]. Third, in countries where health coverage exists, staging delays can be a result of system disruptions, absence of proper diagnosis equipment’s, health providers knowledge gaps, lack of information or complicated bureaucratic steps, and complex or delayed referral mechanisms [36, 38].

Time intervals analysis reveals, within our studied region, an approximate waiting time of 1 month to diagnose the tumor. Also, depending on the tumor type and the need of surgery for curative or palliative purposes, a waiting time of either 1,5 weeks up to a month to receive a first treatment after diagnosis, which according to Cancer Research UK, patients should not wait for more than 28 days for diagnosis and no more than 2 months for first treatment after the tumor first suspicion [39]. These findings hold implications for regional efforts to optimize the management, monitoring, and compliance of referrals, as well as the time taken to complete various stages of the OCP. As such, healthcare professionals and board members can leverage these results to evaluate their practices, identify areas for improvement, and enhance the care quality and overall well-being of oncology patients.

According to several authors, defining a time interval for suspicion-diagnostic-treatment for all types of cancer is complicated due to the heterogenicity of the studies, tumor sites, and healthcare systems [40]. This is confirmed when analyzing comparative studies for the same types of tumors included in this study as waiting times for diagnosis to first treatment tumors like breast, cervix, bladder, gastrointestinal, and lung cancer can vary from 2 weeks up to 2, 3 or even 4 months [41–45].

The section of our work related to the subsequent referral pathways to PHCs indicates that patients are primarily directed towards Central or Reference Oncology Hospitals, although some are referred to the Oncology Department of Regional Hospitals and Public or Private Specialized Clinics, respectively. Moreover, there appears to be a sense of coordination among these institutions.

The referral procedures from PHCs in our study differ with the results obtained by Tsui et al., in which their analysis of primary care and oncology relationships showed case studies of rural PHCs in the United States where most patients are referred to a regional hospital cancer center, thus following a pyramidal healthcare pathway [46]. Another study mapped cancer referral pathways in 10 countries and concluded that by analyzing the schematics and differences in referral processes further research could be prompted towards a better understanding on timeliness of diagnosis and cancer outcomes [47]. Studying local contexts may help identify opportunities to improve care and create best referral practices, as intended by our work.

The PHC staff members stated that most complementary tests for oncology patient are conducted at Private Institutions, the RH and the Central or Reference Oncology Hospital, with few local diagnostic tests except for cervical cancer (which has a different screening process). In contrast, RH staff reported that the majority of their probable diagnosis, diagnostic confirmation and complementary diagnostic tests are done locally, except for breast and cervical cancer (also with different screening processes). None of the hospital responders reported referring all their cancer patients to other institutions, however 45.5% indicate referring some.

Given that the Portuguese healthcare system consists of both public and private sectors, our study found that PHCs participants indicated diagnostic pathways for cancer patients linked to private institutions, which would imply out-of-pocket expenses for patients. These types of expenses have been thoroughly studied for treatments or medical care and until recent years no studies concerning out-of-pocket diagnostic were published [48]. Patients typically incur costs for laboratory and diagnostic services, with additional expenses for biopsies and complications [49–51]. These paid diagnostic pathways, stated by the PHCs key decision makers, could be a solution to lack of diagnostic equipment’s in the region public institutions, different diagnostic and treatment capacities within institutions (PHCs and RHs), complicate referral procedures, and long waiting lists [49].

Regarding the number of responses obtained for the first three questions in this paper (Figs. 3, 4, 5, 6, 7 and 8), our analysis shows higher responses rates from the nursing staff in RHs when compared to PHCs revealing a better notion of the OCP in hospitals compared to the healthcare centers. In a similar manner, the response rate of physicians compared to nurses at PHCs reveals a similar trend.

As stated by Flieger et al., interorganizational coordination in primary care settings and oncology can be influenced by the delegation of roles through coordination as well as the methods for sharing information and its contents. These factors could be a plausible explanation to the lower response rates of the nursing staff at PHCs [52].

As patients move further in the pathway (Tumor suspicion, Diagnosis, Staging and Treatment) the responses from our participants (in Figs. 3, 4, 5, 6, 7 and 8) decreased. The PHCs referral pathways in this paper show patients being referred to other institutions for their treatment and diagnosis leaving the nurses and physicians at PHCs more present in early stages of the pathway. Moreover, once the patient diagnosis and treatment protocols are defined, the RH staff involved with cancer patients decrease depending on their tumor type as this section of the OCP requires more specialized care.

Studies on primary care and oncology relations within healthcare systems indicate several situations regarding healthcare professionals’ involvement and knowledge with their cancer patients. First, Providers might feel discomfort managing care outside their specialties, particularly with patients with poorer prognosis and advanced cancers requiring individualized care. Second, once patients are diagnosed, they might struggle prioritizing primary care until their cancer treatments are concluded, therefore, compromising patient management from primary care settings [53].

The lack of communication between professionals decreases primary care providers knowledge on their patients. As stated before, formal or informal communication systems that allow information transfer and facilitated bidirectional referrals have an advantage of increased rapport between providers in primary care and oncology settings [46]. These settings may be applicable in other contexts of primary care and other specialties co-managing different comorbidities [52]. However, less in understood about these communication settings in oncology, particularly outside the vertically integrated healthcare systems [53].

The reliability analysis of the questionnaire sections shows high level of internal consistency in our scales as determined by the Cronbach alpha values, therefore, measuring the same intended underlying dimension. Since the questionnaires in this study were created specifically for a Portuguese healthcare context, comparison with other studies on the same dimensions measured are not possible. However, studies like the one from Li et al. in China show that applying scales and determining their validity can help healthcare institutions understand proper implementations of CPs [54].

In summary, we determined that patients could initially enter the OCP through PHCs or their RH. At PHCs, diagnostics available are at the regional or reference hospital, or at private institutions. If patients enter through the regional hospital, they are most likely to be diagnosed and treated there depending on hospital capacity. Also, more patients are being referred to National reference hospitals from PHCs than RHs.

It seems that PHCs and central or reference hospitals are well involved at early diagnostic and treatment phases in the patient OCP implying a good coordination and communication between institutions that are not in the same region. The RH could be involved early in the OCP depending on the tumor type; however, it also oversees more patients in advanced stages of their disease. The latter could suggest that some types of tumors are first attended at national reference hospitals before arriving to their RH. Finally, once the patient treatment starts (at the reference or regional hospital) their pathway at the local health unit consists in being followed-up mostly at the RH followed by PHCs.

Finally, the OCP at the studied local health unit appears to focus more on gastrointestinal, lung, and urogenital oncology patients (at a hospital level) with less intervention on breast and cervix tumors which are mostly seen and referred to other institutions from PHCs.

### Strengths and limitations

The main limitation for this study was the collection of information since the first approach was by interview and due to the COVID-19 pandemic questionnaires became the best option for our study. There was low professional availability to first meet and receive a first introduction on the work objectives resulting in longer work periods. Another limitation was the participants of the study were all drawn from the public sector restricting the analysis on differences between the private and public healthcare sectors. Regarding generalization of our results, since our study was in one region our findings may not be the same as in other regions of the Portugal or other countries with different or similar healthcare systems. In terms of strengths, our questions were simple enough that similar analysis can be done in other locations in order to map and design a preliminary clinical pathway for cancer patients depending on the incidence of the tumours for a specific zone. Our study also managed to obtain good hospital response rates and acceptable ones from the PHCs (which could be explained due to less intervention in the OCP); as well as cover the main tumours treated regionally, which strengthen and validate our results. Finally, we explored the healthcare professional perception and not the patients experience regarding cancer pathways [31].

## Conclusions

**Fig. 15:**
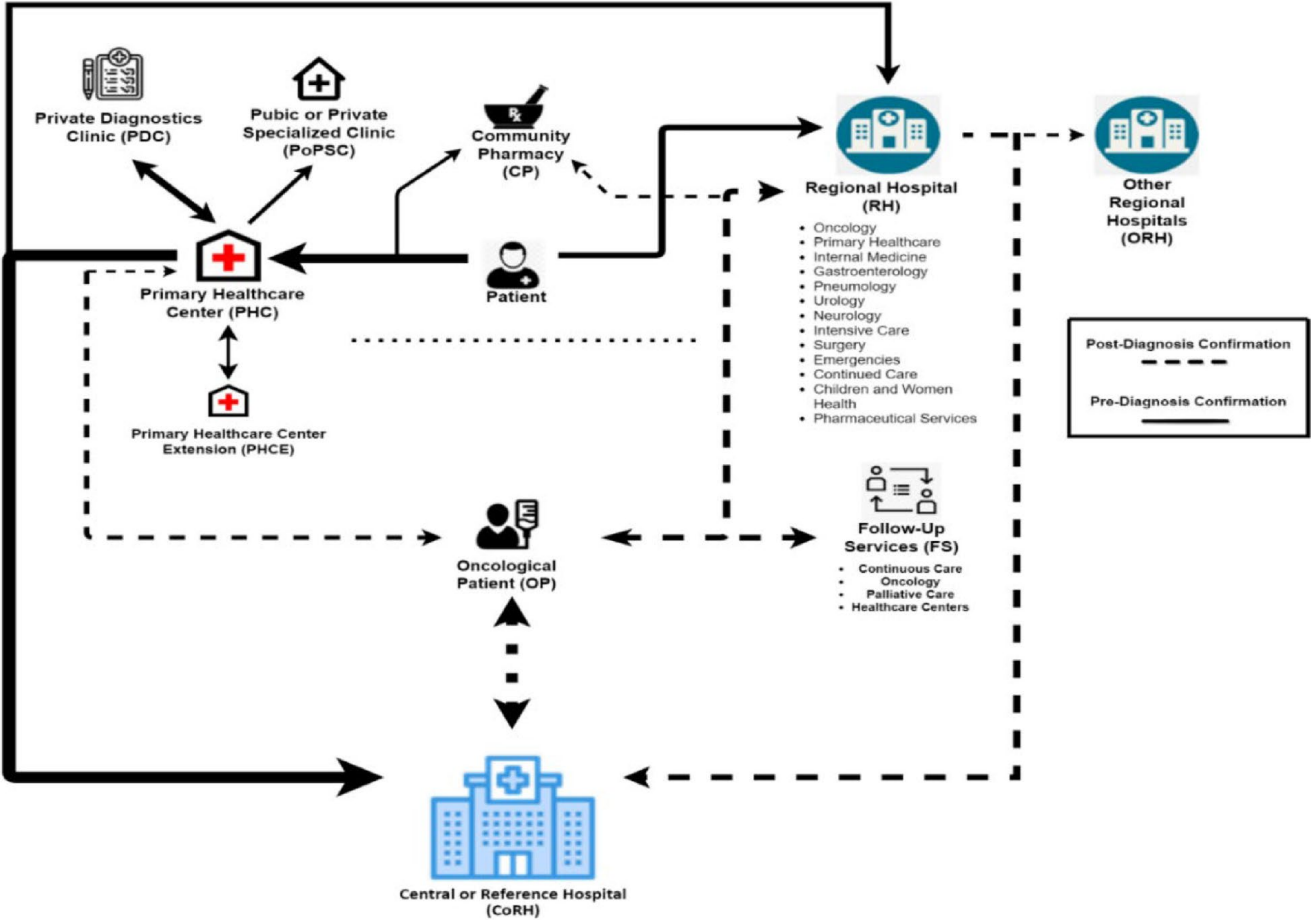
Oncological Clinical Pathway of most patients at the studied local health unit. Patient Pre-Diagnostic Confirmation Pathway order: 1) PHC and PDC, 2) CoRH, 3) RH and/or ORH, 4) PoPSC, 5) CP. OP Post-Diagnostic Confirmation Pathway order: 1) CoRH, 2) RH, 3) FS, 4) PHC, 5) ORH, 6) CP

Our analysis of the oncological clinical pathway at this region reveals how patients transition throughout the healthcare platforms available in the Health System. Questionnaires enable us to understand the real pathway between the different institutions involved (Fig. 15) and allow us to continue this study with a qualitative overview. This work contributes towards the assessment of an initial plan to organize or re-organize an OCP by mapping a design and analyzing the proper functioning between its parts.

### Supplementary Information

**Additional file 1: Annex 1.** Table 1. Examples publications on Clinical Pathways. **Annex 2.** Regional Hospital Questionnaire. **Annex 3.** Primary Healthcare Centres Questionnaire.

## Data Availability

The datasets used and/or analyzed during the current study are available from the corresponding author on reasonable request.

## Abbreviations

CCA: Cholangiocarcinoma
CoRH: Central or Reference Hospital
CPs: Clinical Pathways
FS: Follow-up Services
HCC: Hepatocellular Carcinoma
OCP: Oncological Clinical Pathway
OP: Oncological Patient
ORH: Other Regional Hospital
PDC: Private Diagnostic Clinic
PHC: Primary Healthcare Center
PHCE: Primary Healthcare Center Extension
PoPSC: Public or Private Specialized Clinic
RH: Regional Hospital
