# Supplementary material for: Identification of an oncological clinical pathway through questionnaires to health professionals
Source: BMC Health Serv Res. 2023 Sep 20;23:1011. doi: 10.1186/s12913-023-09964-w (PMC10510255; doi:10.1186/s12913-023-09964-w)
Supplement: Supplementary file 1 — Additional file 1: Annex 1. Table 1. Examples publications on Clinical Pathways. Annex 2. Regional Hospital Questionnaire. Annex 3. Primary Healthcare Centres Questionnaire. [file 12913_2023_9964_MOESM1_ESM.docx]

**Annexes**

**Annex 1.** Table 1. Examples publications on Clinical Pathways.

| Reference | Study Objective |
| --- | --- |
| Dupuis et al. ^13^ | Describe barriers to pediatric cancer symptom management care pathway implementation in 25 pediatric oncology hospitals |
| Chowdhury et al. ^14^ | Present an overview of a pre-hospital acute stroke pathway. Areas to reduce treatment delays and workflow optimization |
| Donal et.al. ^15^ | Describe the development and implementation of an online CP for chronic kidney disease in adults in primary care |
| Alfano et al. ^16^ | Proceedings from the American Cancer Society and ASCO summit to identify steps towards personalized CPs for cancer follow-up |
| Numico et al. ^9^ | Build a unique CP model for all centers in an Italian regional network aiming to make CPs accountable and comparable |
| Van Hoeve et al. ^17^ | Systematic review and meta- analysis aiming to identify and synthesize existing literature on the effects of pathways in oncological care |
| Bencivenga et al. ^18^ | Define the optimal Diagnostic-Therapeutic Pathway to be applied for patients with gastric cancer in the Veneto region |
| Shaw et al. ^19^ | Establish consensus on elements of a draft clinical pathway for anxiety and depression in cancer patients tailored to the Australian context |
| Brenne et al. ^20^ | Develop a CP integrating palliative care and oncology services for hospitalized and home-dwelling palliative cancer patients in a rural region |
| Geerlings et al. ^21^ | Explore staff perspectives on the feasibility and acceptance of strategies to implement a CP for anxiety and depression in cancer patients |
| Ellis et al. ^22^ | Demonstrates that CPs can assist oncology practices decreasing costs and quickly responding to changing treatment paradigms in colorectal cancer |
| Wind et al. ^23^ | Describes the development and results of a benchmark tool for cancer CPs and explore Integrated Practice Units development in cancer centers |
| Gallo et al. ^24^ | Outline a diagnostic and therapeutic CP dedicated to hospitalized patients with diabetes and cancer |
| Seo and Noh ^25^ | Estimate cancer survivors through a principal CP for cancer management and a support system platform that can be used to meet patient needs |
| Solbjør et al. ^26^ | Investigates how patients in Norway experience waiting times within a Cancer CP |

**Annex 2.** Regional Hospital Questionnaire

1. Type of Healthcare Professional:

| *□* | Physician |
| --- | --- |
| *□* | Nurse |

1. What is the stage at which most of your patients arrive (If you have never followed or does not accompany any of the following types of patients and/or type of tumor, please select Not Applicable):

*Check all that apply*

|  | Localized: Initial | Localized: Advanced | Metastatic | Don’t Know | Not Applicable |
| --- | --- | --- | --- | --- | --- |
| *Lung* | *□* | *□* | *□* | *□* | *□* |
| *Rectum* | *□* | *□* | *□* | *□* | *□* |
| *Prostate* | *□* | *□* | *□* | *□* | *□* |
| *Stomach* | *□* | *□* | *□* | *□* | *□* |
| *HCC* | *□* | *□* | *□* | *□* | *□* |
| *Bladder* | *□* | *□* | *□* | *□* | *□* |
| *Intestine* | *□* | *□* | *□* | *□* | *□* |
| *CCA* | *□* | *□* | *□* | *□* | *□* |
| *Breast* | *□* | *□* | *□* | *□* | *□* |
| *Cervix* | *□* | *□* | *□* | *□* | *□* |
| *Other*  *(sPECIFY):* | *□* | *□* | *□* | *□* | *□* |

*HCC: Hepatocellular Carcinoma; CCA: Cholangiocarcinoma*

1. Expected time interval between tumor suspicion and Diagnostic confirmation (If you have never followed or does not accompany any of the following types of patients and/or type of tumor, please select Not Applicable):

*Check all that apply*

|  | < 1 Week | < 2 Weeks | < 4 Weeks | > 1 month | Not Applicable |
| --- | --- | --- | --- | --- | --- |
| *Lung* | *□* | *□* | *□* | *□* | *□* |
| *Rectum* | *□* | *□* | *□* | *□* | *□* |
| *Prostate* | *□* | *□* | *□* | *□* | *□* |
| *Stomach* | *□* | *□* | *□* | *□* | *□* |
| *HCC* | *□* | *□* | *□* | *□* | *□* |
| *Bladder* | *□* | *□* | *□* | *□* | *□* |
| *Intestine* | *□* | *□* | *□* | *□* | *□* |
| *CCA* | *□* | *□* | *□* | *□* | *□* |
| *Breast* | *□* | *□* | *□* | *□* | *□* |
| *Cervix* | *□* | *□* | *□* | *□* | *□* |
| *Other sPECIFY:* | *□* | *□* | *□* | *□* | *□* |

*HCC: Hepatocellular Carcinoma; CCA: Cholangiocarcinoma*

1. Expected time interval between tumor Diagnosis confirmation and First Treatment (If you have never followed or does not accompany any of the following types of patients and/or type of tumor, please select Not Applicable or N/A):

*Check all that apply*

|  | < 1 Week | < 2 Weeks | < 4 Weeks | > 1 month | Not Applicable |
| --- | --- | --- | --- | --- | --- |
| *Lung* | *□* | *□* | *□* | *□* | *□* |
| *Rectum* | *□* | *□* | *□* | *□* | *□* |
| *Prostate* | *□* | *□* | *□* | *□* | *□* |
| *Stomach* | *□* | *□* | *□* | *□* | *□* |
| *HCC* | *□* | *□* | *□* | *□* | *□* |
| *Bladder* | *□* | *□* | *□* | *□* | *□* |
| *Intestine* | *□* | *□* | *□* | *□* | *□* |
| *CCA* | *□* | *□* | *□* | *□* | *□* |
| *Breast* | *□* | *□* | *□* | *□* | *□* |
| *Cervix* | *□* | *□* | *□* | *□* | *□* |
| *Other sPECIFY:* | *□* | *□* | *□* | *□* | *□* |

*HCC: Hepatocellular Carcinoma; CCA: Cholangiocarcinoma*

1. The complementary diagnostic analysis for the different tumors of your patients are performed where? (If you have never followed or do not accompany any of the following type of patient and/or type of tumor, please select Not applicable):

*Check all that apply*

|  | Regional Hospital | Another Hospital | Private Institution | National Reference Oncology Hospital | Primary Healthcare center | Other Institution |  | Not Applicable |
| --- | --- | --- | --- | --- | --- | --- | --- | --- |
| *Lung* | *□* | *□* | *□* | *□* | *□* | *□* |  | *□* |
| *Rectum* | *□* | *□* | *□* | *□* | *□* | *□* |  | *□* |
| *Prostate* | *□* | *□* | *□* | *□* | *□* | *□* |  | *□* |
| *Stomach* | *□* | *□* | *□* | *□* | *□* | *□* |  | *□* |
| *HCC* | *□* | *□* | *□* | *□* | *□* | *□* |  | *□* |
| *Bladder* | *□* | *□* | *□* | *□* | *□* | *□* |  | *□* |
| *Intestine* | *□* | *□* | *□* | *□* | *□* | *□* |  | *□* |
| *CCA* | *□* | *□* | *□* | *□* | *□* | *□* |  | *□* |
| *Breast* | *□* | *□* | *□* | *□* | *□* | *□* |  | *□* |
| *Cervix* | *□* | *□* | *□* | *□* | *□* | *□* |  | *□* |
| *Other sPECIFY:* | *□* | *□* | *□* | *□* | *□* | *□* |  | *□* |

*HCC: Hepatocellular Carcinoma; CCA: Cholangiocarcinoma*

1. Where is the probable tumor diagnosis of most of your patients made at:

*Check all that apply*

|  | NONE | SOME | MANY | ALL |
| --- | --- | --- | --- | --- |
| Primary Healthcare center | *□* | *□* | *□* | *□* |
| *pRIVATE cLINIC* | *□* | *□* | *□* | *□* |
| *REGIONAL HOSPITAL* | *□* | *□* | *□* | *□* |
| *OTHER* | *□* | *□* | *□* | *□* |

1. Where is the diagnostic confirmation of most of your patients’ tumors made at:

*Check all that apply*

|  | NONE | SOME | MANY | ALL |
| --- | --- | --- | --- | --- |
| *National oncology reference hospital* | *□* | *□* | *□* | *□* |
| *another hospital* | *□* | *□* | *□* | *□* |
| *regional hospital* | *□* | *□* | *□* | *□* |
| *private laboratory* | *□* | *□* | *□* | *□* |
| *OTHER* | *□* | *□* | *□* | *□* |

1. Amount of cancer patients’ referrals to other hospitals:

| *□* | All |
| --- | --- |
| *□* | Some |
| *□* | A few |
| *□* | None |
| *□* | No answer |

1. Is Oncology Patient Follow-up a developed activity in your service:

| *□* | Developed |
| --- | --- |
| *□* | Would like to develop or improve |
| *□* | No Answer |

**Annex 3.** Primary Healthcare Centres Questionnaire

1. Type of Healthcare Professional:

| *□* | Physician |
| --- | --- |
| *□* | Nurse |

1. Do you attend cancer patients in your service:

| *□* | Yes |
| --- | --- |
| *□* | No |
| *□* | Don’t Know |

1. What is the stage at which most of your patients arrive (If you have never followed or does not accompany any of the following types of patients and/or type of tumor, please select Not Applicable):

*Check all that apply*

|  | Localized: Initial | Localized: Advanced | Metastatic | Don’t Know | Not Applicable |
| --- | --- | --- | --- | --- | --- |
| *Lung* | *□* | *□* | *□* | *□* | *□* |
| *Rectum* | *□* | *□* | *□* | *□* | *□* |
| *Prostate* | *□* | *□* | *□* | *□* | *□* |
| *Stomach* | *□* | *□* | *□* | *□* | *□* |
| *HCC* | *□* | *□* | *□* | *□* | *□* |
| *Bladder* | *□* | *□* | *□* | *□* | *□* |
| *Intestine* | *□* | *□* | *□* | *□* | *□* |
| *CCA* | *□* | *□* | *□* | *□* | *□* |
| *Breast* | *□* | *□* | *□* | *□* | *□* |
| *Cervix* | *□* | *□* | *□* | *□* | *□* |
| *Other*  *(sPECIFY):* | *□* | *□* | *□* | *□* | *□* |

*HCC: Hepatocellular Carcinoma; CCA: Cholangiocarcinoma*

1. Expected time interval between tumor suspicion and Diagnostic confirmation (If you have never followed or does not accompany any of the following types of patients and/or type of tumor, please select Not Applicable):

*Check all that apply*

|  | < 1 Week | < 2 Weeks | < 4 Weeks | > 1 month | Not Applicable |
| --- | --- | --- | --- | --- | --- |
| *Lung* | *□* | *□* | *□* | *□* | *□* |
| *Rectum* | *□* | *□* | *□* | *□* | *□* |
| *Prostate* | *□* | *□* | *□* | *□* | *□* |
| *Stomach* | *□* | *□* | *□* | *□* | *□* |
| *HCC* | *□* | *□* | *□* | *□* | *□* |
| *Bladder* | *□* | *□* | *□* | *□* | *□* |
| *Intestine* | *□* | *□* | *□* | *□* | *□* |
| *CCA* | *□* | *□* | *□* | *□* | *□* |
| *Breast* | *□* | *□* | *□* | *□* | *□* |
| *Cervix* | *□* | *□* | *□* | *□* | *□* |
| *Other sPECIFY:* | *□* | *□* | *□* | *□* | *□* |

*HCC: Hepatocellular Carcinoma; CCA: Cholangiocarcinoma*

1. Expected time interval between tumor Diagnosis confirmation and First Treatment (If you have never followed or does not accompany any of the following types of patients and/or type of tumor, please select Not Applicable or N/A):

*Check all that apply*

|  | < 1 Week | < 2 Weeks | < 4 Weeks | > 1 month | Not Applicable |
| --- | --- | --- | --- | --- | --- |
| *Lung* | *□* | *□* | *□* | *□* | *□* |
| *Rectum* | *□* | *□* | *□* | *□* | *□* |
| *Prostate* | *□* | *□* | *□* | *□* | *□* |
| *Stomach* | *□* | *□* | *□* | *□* | *□* |
| *HCC* | *□* | *□* | *□* | *□* | *□* |
| *Bladder* | *□* | *□* | *□* | *□* | *□* |
| *Intestine* | *□* | *□* | *□* | *□* | *□* |
| *CCA* | *□* | *□* | *□* | *□* | *□* |
| *Breast* | *□* | *□* | *□* | *□* | *□* |
| *Cervix* | *□* | *□* | *□* | *□* | *□* |
| *Other sPECIFY:* | *□* | *□* | *□* | *□* | *□* |

*HCC: Hepatocellular Carcinoma; CCA: Cholangiocarcinoma*

1. Does your service carry out diagnostic complementary analysis to confirm tumor suspicion in your patients:

| *□* | Yes |
| --- | --- |
| *□* | No |
| *□* | Don’t Know |

1. The complementary diagnostic analysis for the different tumors of your patients are performed where? (If you have never followed or do not accompany any of the following type of patient and/or type of tumor, please select Not applicable):

*Check all that apply*

|  | Primary Healthcare center | Regional Hospital | Private Institution | National Reference Oncology Hospital | Other Institution | Not Applicable |
| --- | --- | --- | --- | --- | --- | --- |
| *Lung* | *□* | *□* | *□* | *□* | *□* | *□* |
| *Rectum* | *□* | *□* | *□* | *□* | *□* | *□* |
| *Prostate* | *□* | *□* | *□* | *□* | *□* | *□* |
| *Stomach* | *□* | *□* | *□* | *□* | *□* | *□* |
| *HCC* | *□* | *□* | *□* | *□* | *□* | *□* |
| *Bladder* | *□* | *□* | *□* | *□* | *□* | *□* |
| *Intestine* | *□* | *□* | *□* | *□* | *□* | *□* |
| *CCA* | *□* | *□* | *□* | *□* | *□* | *□* |
| *Breast* | *□* | *□* | *□* | *□* | *□* | *□* |
| *Cervix* | *□* | *□* | *□* | *□* | *□* | *□* |
| *Other sPECIFY:* | *□* | *□* | *□* | *□* | *□* | *□* |

*HCC: Hepatocellular Carcinoma; CCA: Cholangiocarcinoma*

1. Once the tumor diagnosis or suspicion is confirmed, where is the patient referred to:

*Check all that apply*

| *□* | Regional Hospital Oncology Department |
| --- | --- |
| *□* | National Oncology Reference Hospital |
| *□* | Public or Private |
| *□* | Other |

1. Is there any type of coordination between your Primary Healthcare Center and the institutions in question 8:

| *□* | Yes |
| --- | --- |
| *□* | No |

1. What is the level of coordination between your Primary Healthcare Center and the institutions in question 8: (PHCs)

| *□* | Excellent |
| --- | --- |
| *□* | Good |
| *□* | Reasonable |
| *□* | Insufficient |

1. Is Oncology Patient Follow-up a developed activity in your service:

| *□* | Developed |
| --- | --- |
| *□* | Would like to develop or improve |
| *□* | No Answer |
